# GeoH2 model: Geospatial cost optimization of green hydrogen production including storage and transportation

**DOI:** 10.1016/j.mex.2024.102660

**Published:** 2024-03-12

**Authors:** Claire Halloran, Alycia Leonard, Nicholas Salmon, Leander Müller, Stephanie Hirmer

**Affiliations:** Department of Engineering Science, University of Oxford, Parks Road, OX1 3PJ, United Kingdom

**Keywords:** GIS, Ammonia, Levelized cost of hydrogen, Renewable energy, Power-to-X, GeoH2

## Abstract

This paper presents GeoH2, a geospatial model that optimizes the cost of green hydrogen production, storage, transport, and conversion. This model calculates the cost of producing green hydrogen in a specified location to meet demand in another location by:

• Optimizing hydrogen conversion and transport from production site to demand site
• Optimizing green hydrogen production and storage based on spatially-specific wind and solar generation temporal availability

This method allows users to map production costs throughout a region to identify the lowest-cost location of green hydrogen production to meet demand using a specified end-state for transportation and storage (i.e., pressurized hydrogen, ammonia, or liquefied hydrogen). These modeled costs can be compared to current or projected prices for energy and chemical feedstock in the region to assess the cost-competitiveness of green hydrogen. The model is designed to run at a country or regional scale. A case study application is provided for the context of Namibia.

## Graphical abstract

**Figure:**
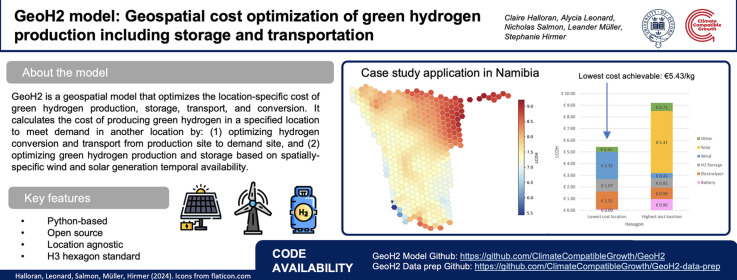

Specifications table

**Table:** 

Subject area:	Energy
More specific subject area:	Energy system modelling
Name of your method:	GeoH2
Name and reference of original method:	This method is an improved and adapted iteration upon work by Müller et al [1]. Müller, L. A., Leonard, A., Trotter, P. A., & Hirmer, S. (2023). Green hydrogen production and use in low-and middle-income countries: A least-cost geospatial modelling approach applied to Kenya. Applied Energy, 343, 121219.
Resource availability:	This method is implemented open-source in Python at the project GitHub repository: https://github.com/ClimateCompatibleGrowth/GeoH2. The spatial data pre-processing toolkit for this method is available at https://github.com/ClimateCompatibleGrowth/GeoH2-data-prep.

## Motivation

This paper presents GeoH2, a geospatial green hydrogen model which optimizes the production, storage, transport, and conversion of green hydrogen while accounting for the location of production and demand. It allows users to identify the lowest-cost location to construct greenfield, non-grid-tied hydrogen production plants to meet a defined temporal demand profile using an optimised mix of wind, solar, battery storage, and hydrogen storage.

Unlike existing commercial models, which do not account for storage and assume that demand is either completely flexible [2] or can be co-optimised with production [3], this model integrates the practical constraints of storage and consistent supply necessary to facilitate industrial use of hydrogen. As many industrial processes require a consistent hydrogen input, this creates a far more realistic cost estimate than models which assume flexible loading.

This model builds upon a preliminary implementation in Kenya produced by Müller et al [1] in two key ways. First, whereas the original selected either wind or solar only, this version optimises both solar and wind in combination. It allows for hybrid system configurations and also co-optimises all other plant equipment and storage in this process. Second, whereas the first iteration was purpose-built for Kenya, this version is made location-agnostic. Indeed, GeoH2 is now built for global applicability. It can be utilised in any user-specified area of interest (AOI) so long as input data for the specified location are added to the user-friendly input data sheets before running the model, and spatial data files for the area of interest are pre-processed. Spatial data for any area of interest can be downloaded and prepared as described in the ‘Method details’ section.

The GeoH2 model, including input data sheet templates, is made open source at the following Github repository: https://github.com/ClimateCompatibleGrowth/GeoH2. A helper toolkit for spatial data pre-processing is available at https://github.com/ClimateCompatibleGrowth/GeoH2-data-prep.

## Method details

This section presents the basic workflow of steps to apply the GeoH2 model. It then goes through the theory behind each stage in detail.

### Workflow summary

To use the GeoH2 model, the user can take the following steps. While this list is intended as a quick-reference for model usage, the full details are presented on each attribute are presented in the following subsections; please refer to these as needed. Step 1 uses the GeoH2-data-prep library, and steps 2-9 use the GeoH2 library. Both libraries are openly available under a CC-BY-4.0 license.

1. **Pre-process spatial data**: Run the spatial_data_prep.py to convert raw data to common coordinate reference systems, file types, and naming conventions. Then, use the workflow.py script to interface with the GLAES package to constrain land availability in the AOI. Next, use the Country_config.yml file to interface with the SPIDER package to aggregate spatial data into H3 hexagons. Finally, combine the GLAES and SPIDER results using combine_glaes_spider.py. This generates a hexagon file ready for use in GeoH2. More details are available in the README.md file of the GeoH2-data-prep library. See Section ‘Spatial pre-processing’ for further details on this step.
2. **Specify input parameters**: Specify input parameters in the Excel files found in the Parameters folder. These include technology availability, demand magnitude and location, country-specific costs and interest rates, transport, conversion, and weather data. See Section ‘Parameter specification’ for further details on this step.
3. **Assign country to hexagons**: Run the assign_country.py script to assign country-specific interest rates, technology lifetimes, and heat and electricity prices from country_parameters.xlsx to the input hexagons.
4. **Download weather data**: Run the get_weather_data.py script to download historical weather data for the AOI and time period of interest specified in the weather parameters file weather_parameters.xlsx. See Section ‘Download weather data’ for further details on this step.
5. **Optimize transport and conversion**: Run the optimize_transport_and_conversion.py script to calculate the cost of the optimal hydrogen transportation and conversion strategy from each hexagon to each demand center using road transport and pipelines based on parameters from technology_parameters.xlsx, demand_parameters.xlsx, and country_parameters.xlsx. See Section ‘Optimize transport and conversion’ for further details on this step.
6. **Optimize green hydrogen plant**: Run the optimize_hydrogen_plant.py script to design a green hydrogen plant to meet hydrogen demand for each demand center and for each transportation mode. Parameters are specified in the Basic_H2_plant folder, investment_parameters.xlsx, and demand_parameters.xlsx. See Section ‘Optimize green hydrogen plant’ for further details on this step.
7. **Calculate water costs** Run the water_cost.py script to calculate ocean and freshwater water costs for hydrogen production in each hexagon using technology_parameters.xlsx and country_parameters.xlsx. See Section ‘Water costs’ for further details on this step.
8. **Calculate and visualise total hydrogen costs**: Run the total_hydrogen_cost.py script to find the lowest-cost method of producing, transporting, and converting hydrogen in each hexagon for each demand center by combining the results of the optimize_transport_and_conversion.py, optimize_hydrogen_plant.py, and water_cost.py scripts. The costs_by_component.py script can be run after total_hydrogen_cost.py to split the levelized cost of hydrogen (LCOH) into cost components associated to different technologies (e.g., electrolyzer, wind, solar). Run the map_costs.py script to produce maps illustrating the results over the AOI. See Section ‘Total hydrogen costs’ for further details on this step.

### Spatial pre-processing

Before running GeoH2, spatial input data for the AOI must be processed into a single GeoJSON file following the H3 hexagon standard [4]. Spatial input data are required to (1) constrain land availability for renewable energy infrastructure, and (2) calculate costs associated with transport and water supply in each hexagon. While the land availability constraints can be customized (i.e., more or less constraints can be included based on the user’s preference), final hexagons must include a distance to water bodies, waterways, and road networks to enable the transport and water calculations. As such, the essential spatial input data are: country boundaries, coastlines, waterways, water bodies, and roads. We also suggest including land availability constraints based on land cover classifications and protected areas, though these can be omitted or changed if desired. These data can be downloaded from the sources suggested in Table 1.

**Table 1:** Key spatial data inputs and recommended sources for GeoH2.

Data	Source
Coastlines	Global Oceans and Seas [5]
Country boundaries	Natural Earth [6]
Land cover	Corine Land Cover (CLC) [7]
Water bodies	OpenStreetMap [8]
Waterways	OpenStreetMap [8]
Roads	OpenStreetMap [8]
Protected areas	OpenStreetMap [8]

The GeoH2-data-prep library facilitates spatial input data preparation [9]. Alongside performing conversions to common coordinate reference systems, file types, and naming conventions, this library interfaces with the Geospatial Land Availability for Energy Systems (GLAES) [10] and Spatially Integrated Development of Energy and Resources (SPIDER) [11] packages.

The GLAES library is used to constrain land availability throughout the AOI in order to determine the maximum number of wind and solar installations which are theoretically possible in each hexagon. An example GLAES script (i.e., workflow.py) is provided in GeoH2-data-prep. This example constrains land availability based on land cover types obtained from Corine Land Cover (CLC) and distance from the coast and protected areas using 100 m resolution. It allocates 4 MW National Renewable Energy Laboratory reference turbines [12] and 100 MWp solar photovoltaic (PV) plants in the available area. For wind installations, this example excludes coastlines, protected areas, herbaceous wetlands, and permanent water bodies. For solar PV, it also excludes agricultural areas. It furthermore excludes a 250 m buffer around coastlines and protected areas. Note that this is just an example to illustrate GLAES usage. The resolution, turbine size, solar plant size, exclusion types, and buffers are project specific. The sample file must be customized to match the types of infrastructure available and the land constraints which are necessary in the AOI.

The script saves the wind turbine and PV plant placements as.SHP files, and the excluded area for both wind and PV as.TIF files. The constraints on land availability can be customized by editing the workflow.py script based on user preferences.

Next, the SPIDER library is used to generate H3 hexagons which tile across the AOI. These are the basis for the GeoH2 input spatial dataset. SPIDER is run using its own command line interface (CLI) – the user provides a configuration file in the CLI which specifies the features of the hexagons to be created. Details on running the CLI are available in the SPIDER documentation [13]. The GeoH2-data-prep library provides a SPIDER configuration file (Country_config.yml) which can be used with the SPIDER CLI to generate the hexagons required for GeoH2. This configuration specifies level four H3 resolution and includes the distance from each hexagon to the nearest ocean, waterway, water body, and road as attributes. While the distance attributes must not be changed, as they are essential to processes in GeoH2, the H3 resolution can be changed to suit the user’s needs. Note that if a smaller hexagon size is used, this will increase the GeoH2 run-time.

Finally, the results of GLAES and SPIDER (i.e., the theoretical turbine/PV placements and the hexagons with distance attributes) are combined to form the final GeoH2 input file. The combine_glaes_spider.py script counts the number of turbines and the number of PV plants per hexagon and appends this number to each hexagon, producing a final GeoJSON ready for use in the GeoH2 model.

### Parameter specification

Along with the spatial input file, a number of technical and economic parameters are inputs for the GeoH2 model. Input parameters include annual hydrogen demand, infrastructure prices, and the cost of capital for a snapshot in time. These parameters are specified in a set of Excel files in the Parameters folder in the GeoH2 library.

The demand parameters file (demand_parameters.xlsx) defines a list of demand centers. The latitude and longitude of each demand center and the annual demand (in terms of hydrogen mass) and desired hydrogen state (i.e., 500 bar H_2_, liquid H_2_, or NH_3_) must be specified. If multiple forms of hydrogen are demanded in one location, the demand center name must be different (e.g., Nairobi H_2_ and Nairobi NH_3_), as duplicate demand center names will cause problems in the model.

The country parameters file (country_parameters.xlsx) includes country- and technology-specific interest rates, heat and electricity costs, and asset lifetimes. These must be added for all countries in the AOI.

The weather parameters file (weather_parameters.xlsx) includes the geographic area, defined with a latitude-longitude bounding box, and temporal range to download historical weather data from the European Centre for Medium-Range Weather Forecasts Reanalysis v5 (ERA5). This dataset is used to calculate wind and solar generation potential across the AOI. At least a year is recommended for the temporal range to capture seasonal variation in renewable potential, but longer time periods will increase computation time for optimizing the hydrogen plant design.

The other parameters files specify the water, transport, and conversion requirements. The technology parameters file (technology_parameters.xlsx) specifies costs and energy densities for water processing and road infrastructure, water demand, and whether road and hydrogen pipeline construction is allowed. The pipeline parameters file (pipeline_parameters.xlsx) includes the price, capacity, and lifetime data for different sizes of hydrogen pipelines. The transport parameters file (transport_parameters.xlsx) includes the parameters related to road transport of hydrogen, including truck speed, cost, lifetime, and capacity. The conversion parameters file (conversion_parameters.xlsx) includes parameters related to converting between states of hydrogen.

Additionally, in the Basic H2 plant folder, there are several comma-separated values (CSV) files containing the global parameters for optimizing the hydrogen plant design. All power units are MW and all energy units are MWh. For more information on these parameters, refer to the PyPSA documentation [14].

### Download weather data

The get_weather_data.py script downloads weather data for the AOI from the ERA5 dataset [15] using the atlite [16] library. These data are used to model historical wind and solar generation potential and inform the hydrogen plant optimization.

The ERA5 dataset is available at an hourly time resolution from 1940 to present and approximately 30 by 30 km spatial resolution. It is freely available to download for registered users. As such, GeoH2 users will need to register at the Copernicus Climate Data Store and set up their API key before running this script.

### Optimize transport and conversion

The optimize_transport_and_conversion.py script calculates the lowest-cost hydrogen transport strategy from every hexagon to every demand center. The transport strategy includes whether to transport the hydrogen by truck or by pipeline, and what form of hydrogen (i.e., 500 bar hydrogen, liquid hydrogen, liquid organic hydrogen carriers (LOHC), or ammonia) to transport. Total transportation and conversion costs include road or pipeline construction, transportation operating costs, conversion to the form used for transportation, and conversion of the transported form of hydrogen to the form required at the demand center.

First, the transport distance is calculated. For each hexagon, the distance from the centroid of the hexagon to each demand center is calculated. If the demand center is within the hexagon, only conversion costs to the final demand state are included in transportation and conversion costs. If the demand center is outside the hexagon, this distance is used to calculate transport costs.

Next, road transport costs are calculated. If road construction is allowed, the cost of extending the nearest road is calculated for each off-road hexagon based on the distance to the nearest road. Otherwise, trucking is not an option for transportation for hexagons which do not have access to roads. The number of trucks and trailers needed is calculated based on total hydrogen demand, distance to the demand center, and average truck speed assuming full-time truck use. The model calculates trucking costs assuming that the trucks travel on the shortest possible path to the demand center, rather than calculating road distance. Trucking costs include diesel price, driver wages, truck capital and operational expenses, and trailer capital and operational expenses.

Finally, if pipeline construction is allowed, the cost of pipeline construction from every hexagon to each demand center is calculated. Otherwise, pipeline transportation is not an option for any hexagon. Pipeline costs per kilometer and compressor costs are defined for three sizes of pipeline based on their capacity: small (up to 1.2 GW), medium (1.2 to 4.7 GW), and large (4.7 to 13 GW).

### Optimize green hydrogen plant

The optimize_hydrogen_plant.py script optimizes investment and operation of an off-grid green hydrogen plant to meet a specific demand. The PyPSA library [14] is used to optimize the capacity of solar PV, onshore wind, electrolyzer, lithium-ion batteries, and compressed hydrogen storage to produce green hydrogen to meet the specified demand. Note that a solver which is compatible with PyPSA (e.g., Cbc, Gurobi) must be installed to complete this step.

Weather data is converted to hourly onshore wind and solar PV potential using atlite [16]. Onshore wind generation potential is calculated using 100 m wind speed, and forecast surface roughness, the aerodynamic roughness length. Publicly-available wind power curves can be used within atlite or custom power curves can be specified. Solar PV potential is calculated using top of atmosphere incident solar radiation, direct and diffuse influx, albedo, and air temperature at 2 m height to calculate panel efficiency. Crystalline silicon solar panels are assumed.

Annual hydrogen demand is converted to hourly demand schedules based on the transport methods considered. For pipeline transport, constant hourly demand is assumed. For trucking transport, an hourly demand profile is created based on the number of trucks arriving at the plant each hour to achieve a roughly constant delivery schedule at the demand center. The trucking demand schedule is designed to achieve no wait time for trucks to fill with hydrogen.

For each hexagon and demand center pair, hydrogen production costs to meet the pipeline and trucking transportation demand profiles and the optimal capacity of several key components are saved.

### 2.7 Water costs

Water costs for the optimised hydrogen plant are calculated using the water_cost.py script. Both freshwater and ocean water options are considered. Treatment costs for each are defined based on the different energy requirements for each source (e.g., to account for desalination in the ocean case). The cost to transport the required demand of water to each hexagon from the nearest available source of each type is also considered in the total water cost. The lowest of the two options is taken as the final water cost, and this is exported as a GeoJSON file (hex_water.geojson).

### Total hydrogen costs

The optimized total hydrogen cost is determined in the total_hydrogen_cost.py script. This total includes production costs, transport costs (including infrastructure construction costs if needed), conversion costs, and water costs. Costs are calculated for both trucking and pipeline transport options. Both are saved, and the lowest of the two is stored as the final optimal lowest cost. All results are saved to a GeoJSON file (hex_total_cost.GeoJSON). Using the cost_by_component.py script, these results can be broken down by technology component. This script provides a file hex_cost_components.geojson with the capacities, capital costs, and overall contribution of each technology towards LCOH for each hexagon.

The results can be visualized using map_costs.py. This script produces heatmaps for each cost component saved to the GeoJSON hex_total_cost.GeoJSON for quick visualisation.

## Case study

This model is demonstrated using a case study of green hydrogen production in Namibia. This case study simulates a hydrogen demand of 60.6 kT/year to be delivered to Lüderitz port (-26.64, 15.14) as green ammonia for export. The demand level is the median planned capacity of those planned projects in the IEA hydrogen database which are: (a) located in continental sub-Saharan Africa, (b) plan to use wind or solar to produce hydrogen via electrolysis, and (c) are intended to go online by 2030 [17]. Interest rates for both production plant technologies and infrastructures are set at 6% for comparability with the IEA levelised cost of hydrogen maps [18]. The case study uses 481 H4-level hexagons. Wetland, built-up areas, and water bodies are excluded from available land for generation/plant construction. The area within 250 m of coastlines or protected areas is also excluded. PV is excluded from agricultural areas. All input files used in this case study, including capital cost assumptions and technical parameters, are made available in the Appendix.

**Fig. 1:**
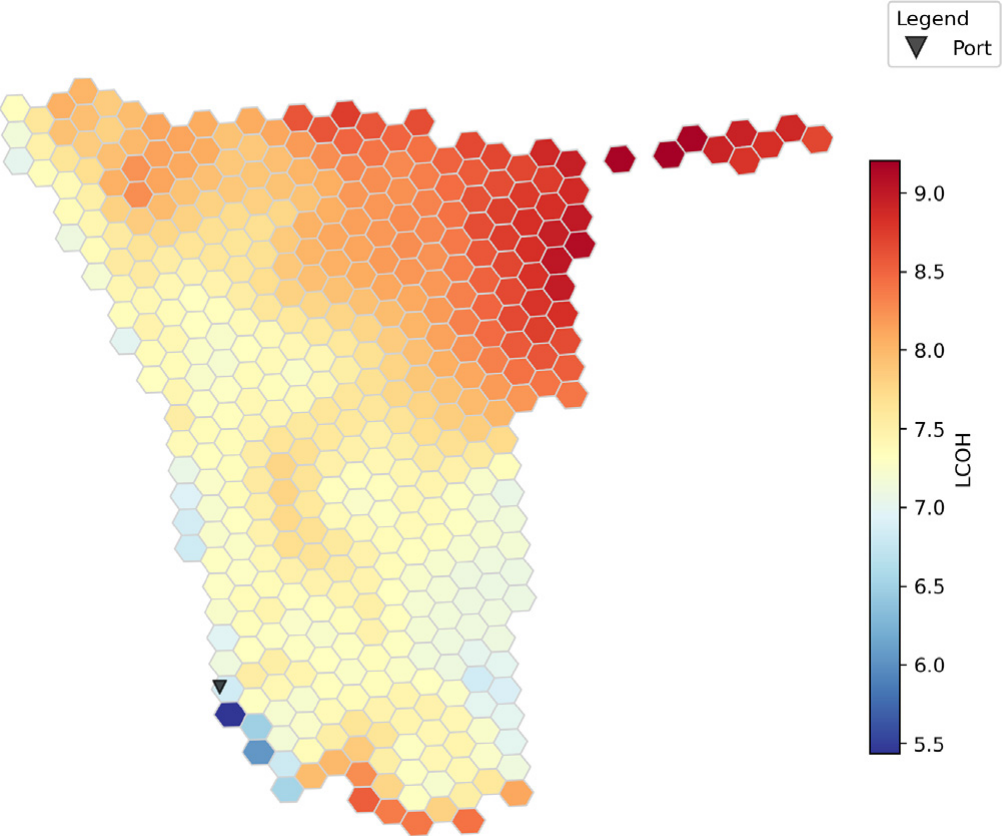
The levelized cost of hydrogen (LCOH) in € /kg for the case study in Namibia.

The LCOH to meet this demand in different locations throughout Namibia is shown in Fig. 1. The costs range between € 5.43 and € 9.21 per kilogram of green hydrogen arriving at Lüderitz. In all hexagons, pipeline transport is the lowest-cost option.

**Fig. 2:**
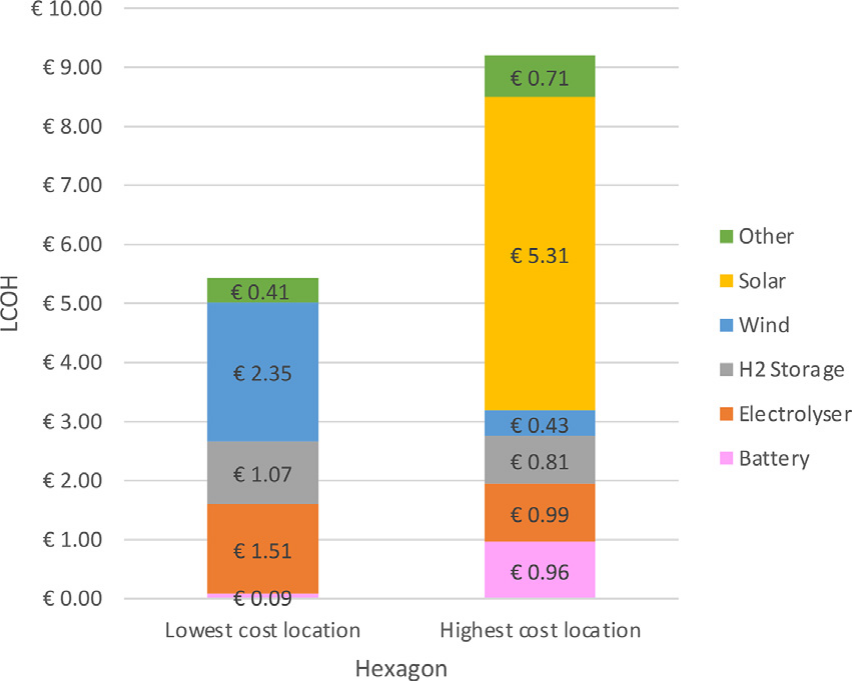
Cost breakdown for the lowest- and highest-cost hexagons for green hydrogen production in Namibia. Note that the costs of transport and water processing are included in the ”Other” segment.

The LCOH for the lowest- and highest-cost production locations are broken down into cost components in Fig. 2. In the lowest-cost location, wind turbines produce all the electricity for electrolysis and very little battery storage is necessary. In the highest-cost location, a large capacity of solar PV as well as wind turbines are needed to produce electricity, and additional battery storage is required because of diurnal variation in solar generation.

## Method validation

Comparing the results of the GeoH2 model with other green hydrogen costs projections validates that the model produces high-end but reasonable cost estimates. The lowest LCOH in the Namibian case study as presented in this paper (i.e., € 5.43 per kg) closely aligns with the estimated LCOH of € 5.13 per kg reported in [19]. Additionally, the Jülich Forschungszentrum projects a marginally lower minmum LCOH of € 4.17 per kg, with an average € 5.04 per kg. This again aligns well with the estimates produced in this case.

However, more optimistic estimates for the future also exist. For instance, the Fraunhofer Institute for Solar Energy Systems projects € 1.13 per kg for 2030 [3]. Similarly, the International Energy Agency projects low-end costs of $2.50 per kg in Namibia by 2030 [18]. Finally, the Namibia Investment Promotion and Development Board, an organization focused on driving investment into Namibia, estimates that $1.50 per kg will be feasible [20]. However, the assumptions underlying these optimistic estimates remain largely unclear.

This points to the larger problem of opaqueness in current hydrogen modelling, for both models which align with GeoH2 results and those which are more optimistic. While the authors would be interested in assessing the influence of different cost and technical assumption in causing the cost variation across models, this is not possible as other models largely do not make their methods and assumptions transparent and accessible.

The strong spatial variation and generally high-end cost projections resulting from the GeoH2 model presented in this paper are unsurprising, as the model is programmed to require a constant hydrogen supply by default. This approach was adopted because many industrial processes necessitate a consistent supply of hydrogen, leading to the assumption of constant hourly demand and resulting in a realistic, albeit high-end, estimate of hydrogen costs. This assumption therefore increases the costs of battery storage, compressed hydrogen storage, and renewable oversizing which enable the consistent supply over time. In contrast, other models make the liberal assumption of fully flexible hydrogen production [2] or leverage genetic algorithms to co-optimise the production profile for least costs [3]. They thus provide a lower bound of hydrogen costs that excludes storage and renewable overcapacity costs. The conservative estimates from the GeoH2 model therefore complement analysis by the Hydrogen Council and other to provide a fuller picture of total hydrogen costs accounting for practical constraints and spatial variability.

## Ethics statements

This work does not involve human subjects, animal experiments, or data collected from social media platforms. Ethical best practices were followed in all cases.

## CRediT authorship contribution statement

**Claire Halloran:** Methodology, Software, Data curation, Writing – original draft, Writing – review & editing. **Alycia Leonard:** Methodology, Validation, Data curation, Writing – original draft, Writing – review & editing. **Nicholas Salmon:** Methodology, Resources, Writing – review & editing. **Leander Müller:** Methodology, Software. **Stephanie Hirmer:** Conceptualization, Resources, Writing – review & editing, Supervision, Project administration, Funding acquisition.

## Declaration of competing interest

The authors declare that they have no known competing financial interests or personal relationships that could have appeared to influence the work reported in this paper.

## Figures and Tables

**Table:** 

Hardware	Parameter	Value	Units	Ref.
Solar photovoltaic	Capex	1,470,000	€ /MW	[21]
Wind turbines	Capex	1,580,000	€ /MW	[21]
Hydrogen electrolysis	Capex	1,250,000	€ /MW	[1]
Hydrogen electrolysis	Efficiency	0.59	MWh_H2_/MWh_el_	[22]
Hydrogen compression	Isentropic efficiency	0.051	MWh_el_/MWh_H2_	[1]
Hydrogen storage unloading	Efficiency	1	MWh_H2_/MWh_H2-stored_	Assumption
Battery	Capex	95,000	€ /MW	[23]
Hydrogen storage	Capex	21,700	€ /MWh	[1]

**Table:** 

Process	Parameter	Value	Units	Ref.

500 bar compression	Heat capacity	0.0039444	kWh/kg/K	[24]
500 bar compression	Input temperature	298.15	K	[1]
500 bar compression	Input pressure	25	bar	[1]
500 bar compression	Isentropic exponent	1.402		[24]
500 bar compression	Isentropic efficiency	0.8		[1]
500 bar compression	Compressor lifetime	15	years	[25]
500 bar compression	Compressor capex coefficient	40,035	€ /kg_H2_/day	[25]
500 bar compression	Compressor opex	4	% capex/year	[25]
Hydrogen liquification	Electricity demand	9.93	kWh/kg_H2_	[26]
Hydrogen liquification	Capex quadratic coefficient	-0.0002	€ /(kg_H2_)^2^	[1]
Hydrogen liquification	Capex linear coefficient	1,781.9	€ /kg_H2_	[1]
Hydrogen liquification	Capex constant	300,000,000	€	[1]
Hydrogen liquification	Opex	8	% capex/year	[25]
Hydrogen liquification	Plant lifetime	20	years	[25]
LOHC hydrogenation	Electricity demand	0.35	kWh/kg_H2_	[27]
LOHC hydrogenation	Heat demand	-9	kWh/kg_H2_	[28]
LOHC hydrogenation	Capex coefficient	0.84	kWh/kg_H2_/year	[29]
LOHC hydrogenation	Opex	4	% capex/year	[29]
LOHC hydrogenation	Plant lifetime	25	years	[29]
LOHC hydrogenation	Carrier costs	2	€ /kg_carrier_	[30]
LOHC hydrogenation	Carrier ratio	16.1	kg_carrier_/kg_H2_	[31]
LOHC dehydrogenation	Electricity demand	0.35	kWh/kg_H2_	[27]
LOHC dehydrogenation	Heat demand	12	kWh/kg H2	[28]
LOHC dehydrogenation	Capex coefficient	2.46	kWh/kg_H2_	[29]
LOHC dehydrogenation	Opex	4	% capex/year	[29]
LOHC dehydrogenation	Plant lifetime	25	years	[29]
Ammonia synthesis	Electricity demand	2.809	kWh/kg_H2_	[32]
Ammonia synthesis	Capex coefficient	0.75717	kWh/g_H2_/year	[32]
Ammonia synthesis	Opex	1.5	% capex/year	[29]
Ammonia synthesis	Plant lifetime	25	years	[29]
Ammonia cracking	Heat demand	4.2	kWh/kg_H2_	[27]
Ammonia cracking	Capex coefficient	17,262,450	kWh/g_H2_/hour	[33]
Ammonia cracking	Opex	2	% capex/year	[1]
Ammonia cracking	Plant lifetime	25	years	[1]

**Table:** 

Hardware	Parameter	Value	Units	Ref.

All trucks	Average truck speed	70	km/h	Assumption
All trucks	Working hours	24	h/day	Assumption
All trucks	Diesel price	1.5	€ /L	Assumption
All trucks	Driver wage	2.85	€ /h	[1]
All trucks	Working days	365	days/year	Assumption
All trucks	Max driving distance	160,000	km/year	[1]
All trucks	Truck capex	160,000	€	[34]
All trucks	Truck Opex	12	% capex/year	[34]
All trucks	Diesel consumption	35	L/100 km	[34]
All trucks	Truck lifetime	8	years	[34]
All trucks	Trailer lifetime	12	years	[34]
500 bar hydrogen trailer	Trailer capex	660,000	€	[25]
500 bar hydrogen trailer	Trailer opex	2	% capex/year	[25]
500 bar hydrogen trailer	Trailer capacity	1,100	kg_H2_	[25]
500 bar hydrogen trailer	Loading and unloading time	1.5	hours	[25]
Liquid hydrogen trailer	Trailer capex	860,000	€	[34]
Liquid hydrogen trailer	Trailer opex	2	% capex/year	[34]
Liquid hydrogen trailer	Trailer capacity	4,300	kg_H2_	[34]
Liquid hydrogen trailer	Loading and unloading time	3	hours	[34]
LOHC trailer	Trailer capex	660,000	€	[29]
LOHC trailer	Trailer opex	2	% capex/year	[34]
LOHC trailer	Trailer capacity	1,800	kg_H2_	[34]
LOHC trailer	Loading and unloading time	1.5	hours	[34]
Ammonia trailer	Trailer capex	210,000	€	[29]
Ammonia trailer	Trailer opex	2	% capex/year	[29]
Ammonia trailer	Trailer capacity	2,600	kg_H2_	[29]
Ammonia trailer	Loading and unloading time	1.5	hours	[29]

**Table:** 

Road length	Parameter	Value	Units	Ref.

Short road (<10 km)	Capex	626,478.45	€ /km	[1]
Long road (>10 km)	Capex	481,866.6	€ /km	[1]
All roads	Opex	7,149.7	€ /km/year	[1]

**Table:** 

Pipeline size	Parameter	Value	Units	Ref.

All pipelines	Opex	1.25	% capex/year	[35]
All pipelines	Availability	95	%	[1]
All pipelines	Pipeline lifetime	42.5	years	[35]
All pipelines	Compressor lifetime	24	years	[35]
All pipelines	Electricity demand	0.000614	kWh/kg_H2_/km	[35]
Large pipeline	Maximum capacity	13	GW	[35]
Large pipeline	Pipeline capex	2,800,000	€ /km	[35]
Large pipeline	Compressor capex	620,000	€ /km	[35]
Medium pipeline	Maximum capacity	4.7	GW	[35]
Medium pipeline	Pipeline capex	2,200,000	€ /km	[35]
Medium pipeline	Compressor capex	310,000	€ /km	[35]
Small pipeline	Maximum capacity	1.2	GW	[35]
Small pipeline	Pipeline capex	90,000	€ /km	[35]
Small pipeline	Compressor capex	90,000	€ /km	[35]

**Table:** 

Type	Parameter	Value	Units	Ref.

Freshwater	Treatment electricity demand	0.4	kWh/m^3^ water	[36]
Ocean water	Treatment electricity demand	3.7	kWh/m^3^ water	[37]
All water	Transport cost	0.1	€ /100 km/m^3^ water	[38]
All water	Water specific cost	1.25	€ /m^3^ water	[39]
All water	Water demand	21	L water/kg_H2_	[22]

**Table:** 

Country	Parameter	Value	Units	Ref.

Namibia	Electricity price	0.10465	€ /kWh	[40]
Namibia	Heat price	0.02	€ /kWh	Assumption
Namibia	Solar interest rate	6	%	Assumption
Namibia	Solar lifetime	20	years	Assumption
Namibia	Wind interest rate	6	%	Assumption
Namibia	Wind lifetime	20	years	Assumption
Namibia	Plant interest rate	6	%	Assumption
Namibia	Plant lifetime	20	years	Assumption
Namibia	Infrastructure interest rate	6	%	Assumption
Namibia	Infrastructure lifetime	50	years	[1]

## Data Availability

All data used is available in the appendix and/or the Github repositories linked in-text.

## Appendix A Case study parameter values

**Green hydrogen plant**

**Conversion parameters**

**Trucking**

**Roads**

**Pipelines**

**Water**

**Country-specific parameters**
